# Food Security Monitoring via Mobile Data Collection and Remote Sensing: Results from the Central African Republic

**DOI:** 10.1371/journal.pone.0142030

**Published:** 2015-11-18

**Authors:** Markus Enenkel, Linda See, Mathias Karner, Mònica Álvarez, Edith Rogenhofer, Carme Baraldès-Vallverdú, Candela Lanusse, Núria Salse

**Affiliations:** 1 Vienna University of Technology, Department of Geodesy and Geoinformation, Vienna, Austria; 2 International Institute for Applied Systems Analysis, Laxenburg, Austria; 3 Doctors without Borders/Médecins Sans Frontières (MSF), Barcelona, Spain; 4 Doctors without Borders/ Médecins Sans Frontières (MSF), Vienna, Austria; Cairo University, EGYPT

## Abstract

The Central African Republic is one of the world’s most vulnerable countries, suffering from chronic poverty, violent conflicts and weak disaster resilience. In collaboration with Doctors without Borders/Médecins Sans Frontières (MSF), this study presents a novel approach to collect information about socio-economic vulnerabilities related to malnutrition, access to resources and coping capacities. The first technical test was carried out in the North of the country (sub-prefecture Kabo) in May 2015. All activities were aimed at the investigation of technical feasibility, not at operational data collection, which requires a random sampling strategy. At the core of the study is an open-source Android application named SATIDA COLLECT that facilitates rapid and simple data collection. All assessments were carried out by local MSF staff after they had been trained for one day. Once a mobile network is available, all assessments can easily be uploaded to a database for further processing and trend analysis via MSF in-house software. On one hand, regularly updated food security assessments can complement traditional large-scale surveys, whose completion can take up to eight months. Ideally, this leads to a gain in time for disaster logistics. On the other hand, recording the location of every assessment via the smart phones’ GPS receiver helps to analyze and display the coupling between drought risk and impacts over many years. Although the current situation in the Central African Republic is mostly related to violent conflict it is necessary to consider information about drought risk, because climatic shocks can further disrupt the already vulnerable system. SATIDA COLLECT can easily be adapted to local conditions or other applications, such as the evaluation of vaccination campaigns. Most importantly, it facilitates the standardized collection of information without pen and paper, as well as straightforward sharing of collected data with the MSF headquarters or other aid organizations.

## Introduction

In many regions that are prone to hydro-meteorological disasters, there is generally a distinct coupling between climatic shocks and socio-economic vulnerabilities [1–3]. While droughts and their precursors (e. g. ocean temperature anomalies) can be monitored by satellites, the resulting socio-economic impacts or the drought-independent factors that are related to people’s vulnerabilities/coping capacities remain mostly hidden to the eye of a satellite. Yet, identifying and quantifying the relationship between remotely-sensed drought indicators and the temporal development of people’s livelihoods, their vulnerabilities and their coping capacities is crucial if remote sensing is to be used as an additional basis for decision-making, and not just as a confirmation of already existing conditions as is often the case. For example, very detailed, large-scale crop and food security assessment missions (CFSAM) are carried out by the United Nations FAO/WFP [4]. These missions generally take place during the crisis or post-event and last between four and eight months. While such in-depth assessments are ideal for retrospective documentation of the relief efforts, they do not capture the local conditions as situations evolve on the ground. What is missing is a tool that allows for regular, high frequency assessments throughout the critical periods for food security, i.e. crop growing seasons, with information on satellite-derived drought indicators.

In October 2014 the number of active mobile devices (https://gsmaintelligence.com) exceeded the world population. In sub-Saharan Africa alone, the market is expected to grow from 635 million subscriptions in late 2014 to 930 million by 2019 –double the growth rate compared to the rest of the world [5]. Despite the poor working conditions in which many of these phones are produced, this development has many positive consequences: improvements in people’s ability to operate smart phones, the extension of mobile phone networks and better framework conditions for new applications, such as food security assessments.

This paper presents a mobile solution that will allow humanitarian organizations to carry out regular, high frequency assessments on the ground with timely inputs from satellite-derived drought indicators that can act as both a source of information and a validation tool for these indicators. The outsourcing of data collection and other micro-tasks to a crowd of people is referred to as “crowdsourcing” and was first introduced by Jeff Howe [6]. Crowdsourced tasks have covered a wide range of applications in the past, ranging from non-scientific applications, such as journalism [7], the filling of databases (e. g. online photo portals) to scientific applications, such as software development [8], astronomy [9], public health [10], analyzing global trends based on online queries [11], land use mapping [12,13] or applications related to humanitarian aid, such as crisis mapping [14–16]. Traditionally, the “crowd” is an anonymous group of individuals, which can therefore limit the trust in the data provided. If the data are used as an additional knowledge base for operational actions of humanitarian aid organizations, these limitations can be critical. As a consequence, we focus on “groupsourcing” or “expertsourcing”, i.e. crowdsourcing performed by a known and trained group of individuals, which in this study is to support the early warning capacities of Doctors without Borders/Médecins Sans Frontières (MSF) with regard to food insecurity. This study presents a methodological approach for high frequency food security assessments with smart phones including the advantages and limitations of such an approach and the relevant results from the application of the tool in the Kabo region of the Central African Republic. In addition, we analyze the added-value of the new approach for operational decision-support in a system that links local data collection to satellite-detected climatic shocks.

## Materials and Methods

### 2.1 Study Area

**Fig 1:**
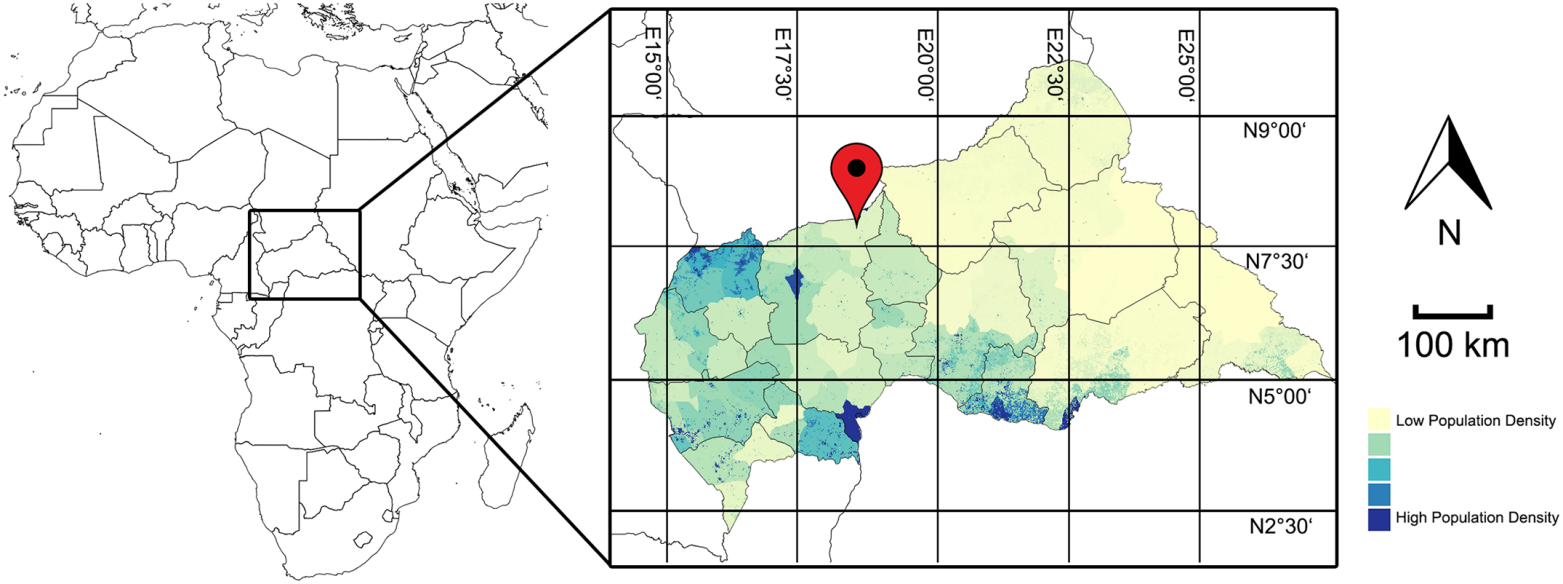
Illustration of the Region of Interest (Kabo) in the Central African Republic (Kabo) and Population Density based on the Worldpop Project (http://www.worldpop.org.uk/).

This study concentrates on the food security assessment that was carried out in the sub-prefecture Kabo (prefecture Ouham) of the Central African Republic (CAR) within a research project named SATIDA (Satellite Technologies for Improved Drought Risk Assessment). Fig 1 illustrates the location of the sparsely populated region of interest in the Central African Republic, which has a surface of roughly 10 km^2^. According to the Global Information and Early Warning System of the UN Food and Agriculture Organizations (FAO GIEWS), out of a total population of 4.6 million people, 1.5 million had been in need of food assistance in 2014. The Integrated Food Security Classification (IPC) level was raised to level 3 (crisis) and 4 (emergency) in many Western parts of the country. A CFSAM report [17] and a special report on crop and food security [18] were published by the UN FAO and WFP in April and October 2014. While the report from April focused on a forecast of the conditions for the 2014 season and the lack of funding for two thirds of the people in need (0.8 million), the latter document highlighted the decrease in crop production (58% lower than pre-crisis average, but 11% higher than 2013), its negative impact on the country’s gross domestic product, an uncovered cereal deficit of at least 57 000 tons for the 2014/2015 season and the urgent need to set up a food security early warning system in the country. By April 2014, FAO announced plans to assist 150 000 households with seeds and farming tools, which could be used due to relatively favourable weather conditions. However, FAO also admitted that funding for only 86 000 households could be secured. As of March 2015, the number of IDP (internally displaced people) was still estimated at roughly 436 000 people, about ten percent of the total population. The Famine Early Warning Systems Network (FEWSNET) provided little recent information about the conditions in the Central African Republic, but generally agreed with the statements of FAO and WFP. In November 2014, FEWSNET [19] estimated the people in need of assistance during the 2015 lean seasons (May to September) at roughly one million people.

While the World Health Assembly aims at reducing the number of stunted children under the age of five by 40% by 2025, the prevalence of stunting in the CAR was still 41% in 2010 [20]. Stunting and other forms of undernutrition do not only mean that children are more susceptible to a variety of chronic diseases. They also affect brain development and subsequently their cognitive abilities, earnings and finally the development of nations.

Acknowledging the above-mentioned factors and the need for humanitarian assistance, mainly caused by the highly volatile security situation, the MSF Operational Centre Barcelona Athens (OCBA) has been present in Kabo since 2006. MSF are running one hospital in Kabo with roughly 165 staff (mainly locals) and three health centres (Gbazara, Farazala and Moyen Sido). Kabo was selected as the region of interest due to the sensitivity of the current (as of May 2015) situation with regard to food security and sufficiently secure working conditions. The study was supported by MSF Austria, MSF-OCBA, the International Institute for Applied Systems Analysis (IIASA) and Vienna University of Technology (TUW).

### 2.2 Methodology

The objective of the field test was to focus on the technical feasibility and user-friendliness of the new approach from the perspective of local MSF community health workers (CHW), not to collect data operationally. As a consequence, all data collected only reflect the reality of households interviewed and do not represent the entire population. It should be noted that, for operational purposes, the heads of the individual “quartiers” (districts) need to be informed to avoid confusion and anxiety among the population that might not be familiar with smart phones. In addition, a random sampling method (e. g. assessments in every 10^th^ house) needs to be applied to guarantee the representativeness of the assessments. This involves asking people to stay at home, which means that households lose half a day for every day business (e. g. agriculture), kindergarten or school. Hence, a sound balance between the frequency of assessments and the disturbance of daily routines is crucial for operational assessments, affecting both the quantity of collected data and the surveys’ acceptance.

During the stay in Kabo we learned that children in five quartiers were regularly (up to weekly) screened for edema, one of the most obvious symptoms of malnutrition, and MUAC (Mid Upper Arm Circumference) measurements were performed. From a human resources perspective, CHWs who are already familiar with the basics of food security assessments provided a convenient basis for the introduction of a new data collection tool. The questionnaires (S1 Table) list all questions that were used in the assessment in French and English. The actual assessments were carried out in the local language Sangho. Most questions were adapted during the course of the fieldwork via an iterative approach based on the understanding of the CHWs, local conditions and vocabulary.

**Fig 2:**
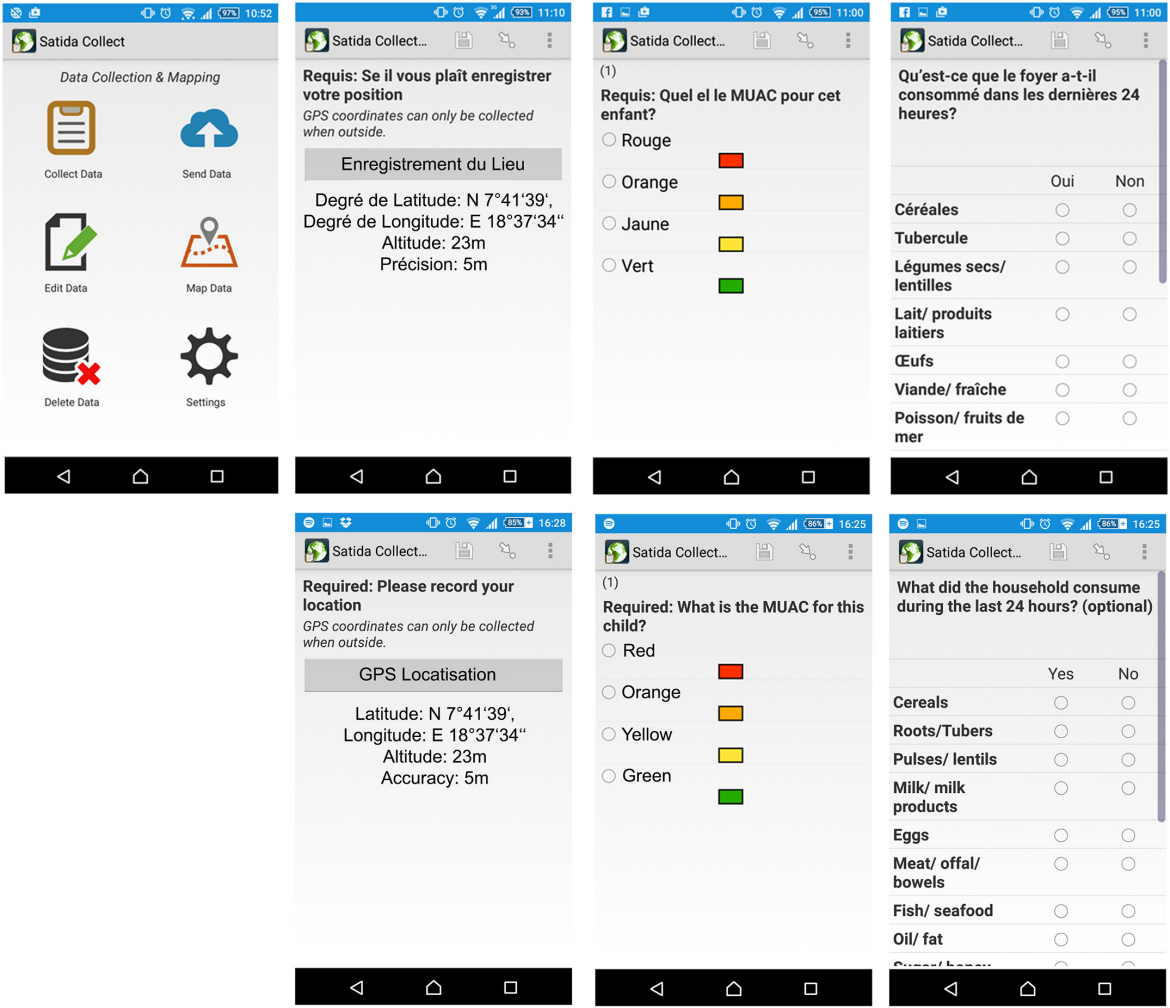
Selected Screenshots of SATIDA COLLECT (start screen, GPS localisation, MUAC measurement, food consumed during last 24 hours) in French and English.

**Fig 3:**
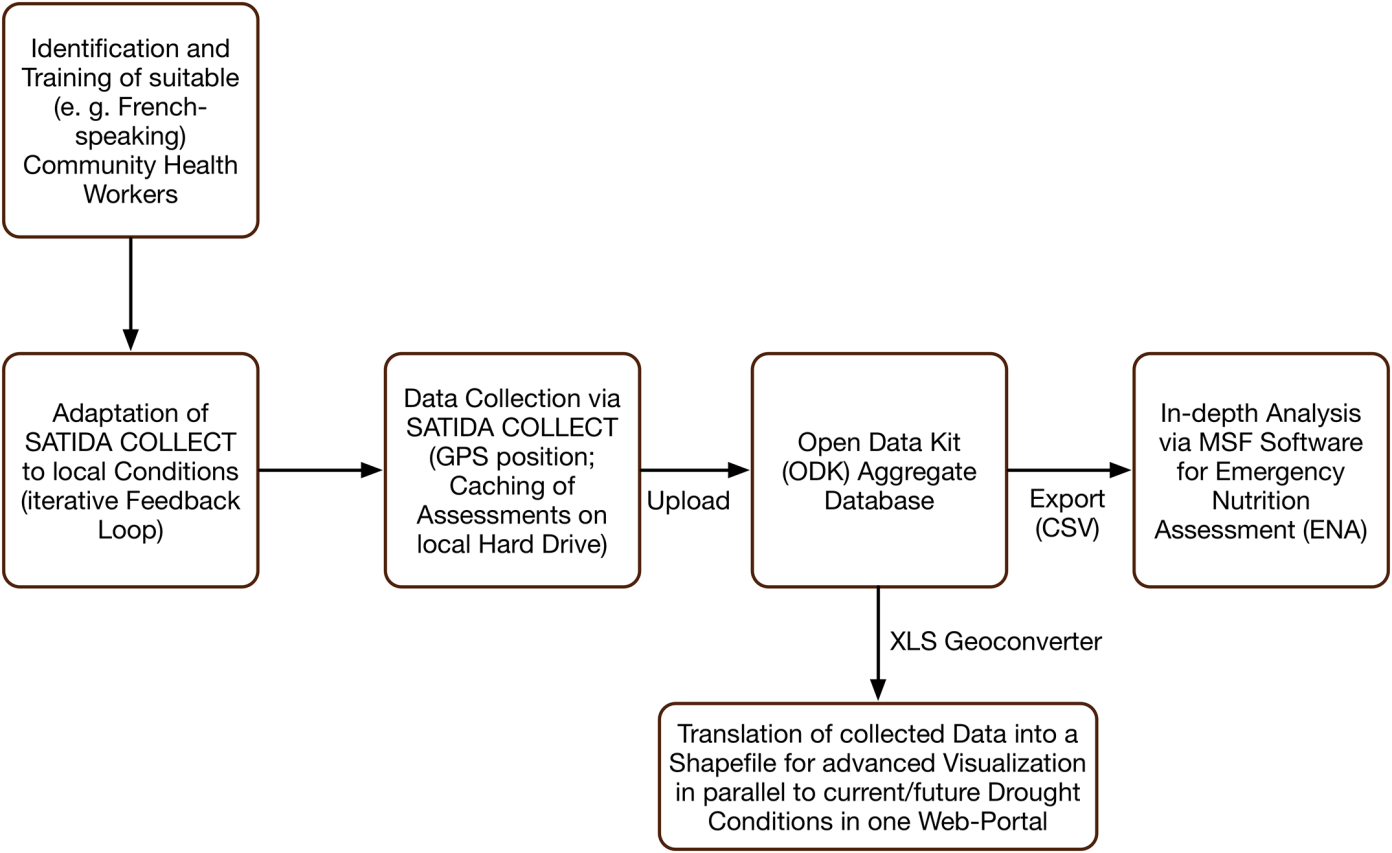
Technical Set-up of SATIDA COLLECT.

The mobile application, SATIDA COLLECT (Fig 2), was built using “Open Data Kit (ODK) aggregrate” [21], a free and open-source toolkit for data collection. GeoODK (http://geoodk.com/) is the spatially-enabled version of ODK. In our set-up (Fig 3), ODK aggregate runs on a Tomcat server with a PostgreSQL database server running in the background. Access to the database can be restricted by a password. Every completed assessment is stored locally (cached) on the phones. Once a connection is available, all completed assessments can be uploaded at once via one click on the phone. This facilitates immediate access to the assessment on the database for (inter)national staff as well as to other aid organizations. In those situations where it is necessary to adapt the questions to changes in conditions or for different applications, users can edit a MS Excel template and convert it into the required xls data format via different file converters (e. g. the Python-based “pyxform” or Nafundi’s XLSForm Converter). Once SATIDA COLLECT is opened, updating the questions on the phone is a matter of three clicks. Finally, we aimed to maximize the interoperability of the collected data with MSF in-house data analysis software for Emergency Nutrition Assessment (ENA). The ENA software enables vital follow-up processing, such as data filtering, cleaning or “denoising”, which will increase their usability [14], as well as trend analysis. This interoperability is guaranteed by the ODK aggregate service providing the data in common file formats such as csv, kml or json, for further processing. For automatic access to the collected data, SATIDA COLLECT provides an application programming interface (API) that allows users to query the data via simple http-requests. In this way, the collected data can, for instance, be displayed on a web viewer in addition to satellite data.

In order to better understand if the food insecurity situation in 2014 was linked to a climatic shock, we analyzed time series of satellite derived rainfall [22,23] and soil moisture [24,25]. The TAMSAT rainfall dataset is well calibrated due to the availability of rainfall gauges close to our region of interest. The soil moisture component is based on a dataset that was developed in the framework of the European Space Agency’s Climate Change Initiative (ESA CCI). We adapted the ESA CCI soil moisture processing chain for the integration of near-real time observations from the Advanced Scatterometer (ASCAT) on-board the MetOp satellites and the Advanced Microwave Scanning Radiometer 2 (AMSR2). This way we could extend the original temporal coverage (1978–2013) to the present. The TAMSAT dataset was resampled to fit the spatial resolution of the ESA CCI soil moisture dataset, which is 0.25 degrees (roughly 28 kilometers). All operations, including the calculation of anomalies based on a climatology from 1992 to 2015, were performed by a toolbox named POETS (Python Open Earth Observation Tools; [26]. The monthly Standardized Precipitation Evapotranspiration Index [27], which is available at a spatial resolution of 0.5 degrees from 1950 to the present, is used as an additional, independent reference. All time series for the Kabo region were extracted via a nearest neighbour search.

### 2.3 Ethics Statement

Prior to the field test the protocol of the study was reviewed and approved by the MSF ethics review board (http://fieldresearch.msf.org/msf/handle/10144/11645). Since the ethical guidelines of the MFS review board do not envisage a written consent, the assessments were carried out after verbal informed consent of the participants. Data are available according to the terms of the MSF Data Sharing Policy. All assessments were subject to strict confidential use. The analysis of anonymized assessments was restricted to academic purposes. With regard to privacy issues it should be noted that we used one field in SATIDA COLLECT to assign a name to each household. However, just like the GPS localisation this field can easily be deactivated for operational purposes if a user decides to work with fully anonymized data from the beginning.

### 2.3 Availability and Licensing

The SATIDA COLLECT application is freely available and adheres to the Open Source Definition (http://opensource.org/docs/osd). You are free to download, share and modify the source code (https://github.com/karner/collect/tree/satida-collect).

## Results and Discussion

The food security assessments were conducted by three CHWs under the supervision of MSF and TUW staff. While attempting to cover large parts of each quartier, households were selected according to the presence of adults. The exact location of each household was recorded via the internal GPS built into the smart phones with a maximum accuracy of four meters—enough to distinguish houses. The CHWs were trained for one day (six hours) and were able to read and speak in French. They were not experienced in the use of smart phones, creating an additional “challenge” (e. g. for entering text, differentiating tapping from swiping, etc.). After the completion of assessments in three quartiers, the CHWs were asked to train three other CHWs. With the exception of one CHW that had problems to understand, interpret and pose questions without prior translation into the local language Sangho, this experiment was successful.

Table 1 lists the key outputs of the assessment for 101 households in 5 quartiers (Kabo 1 and 2, Demona, Bessantoua and Rounga), representing 923 household members in total. On average, households consisted of 9.3 members and consumed less than one meal per day. In line with the WFP and FAO reports, the number of people that joined the households during the last six months was 92 (roughly 10% of all people in the assessment). On average, people estimated that their food reserves would last less than a week. The most widespread coping mechanisms were buying cheaper food (88%) and days without consuming meals at all (77%). 30% of all households had received a general food distribution (GFD), mostly in 2014. If a child suffers from edema this is a clear sign of malnutrition and no additional MUAC (mid upper arm circumference) was carried out. From all 232 tested children, none suffered from edema. Virtually all MUAC measurements were green (normal).

**Table 1:** Key results of the assessment (HH = Household).

**Number of Households (HH)**	101	**Average Number of Meals per HH**	0,9	**HH buying cheaper Food**	88%
**Family members**	923	**Weeks that Food Reserves will last (Average)**	0,8	**Days without Meals**	73%
**Children 6 to 59 Months**	232	**HH consuming Cereals (last 24 hours)**	63%	**HH consuming immature Harvest**	59%
**Births during last 6 Months**	49	**HH consuming Pulses (last 24 h)**	59%	**Reduced Expenditures for Health/Education**	63%
**Deaths during last 6 Months**	56	**HH consuming Fruits (last 24 h)**	65%	**HH received Food Aid**	30% (mostly 2014)
**People joined the Household during last 6 Months**	92	**HH consuming Meat/Fish (last 24 h)**	15%39%	**HH selling poultry**	32%
**People left the Household during last 6 Months**	53	**HH consuming Peanuts (last 24 h)**	63%	**HH selling cattle**	9%

**Fig 4:**
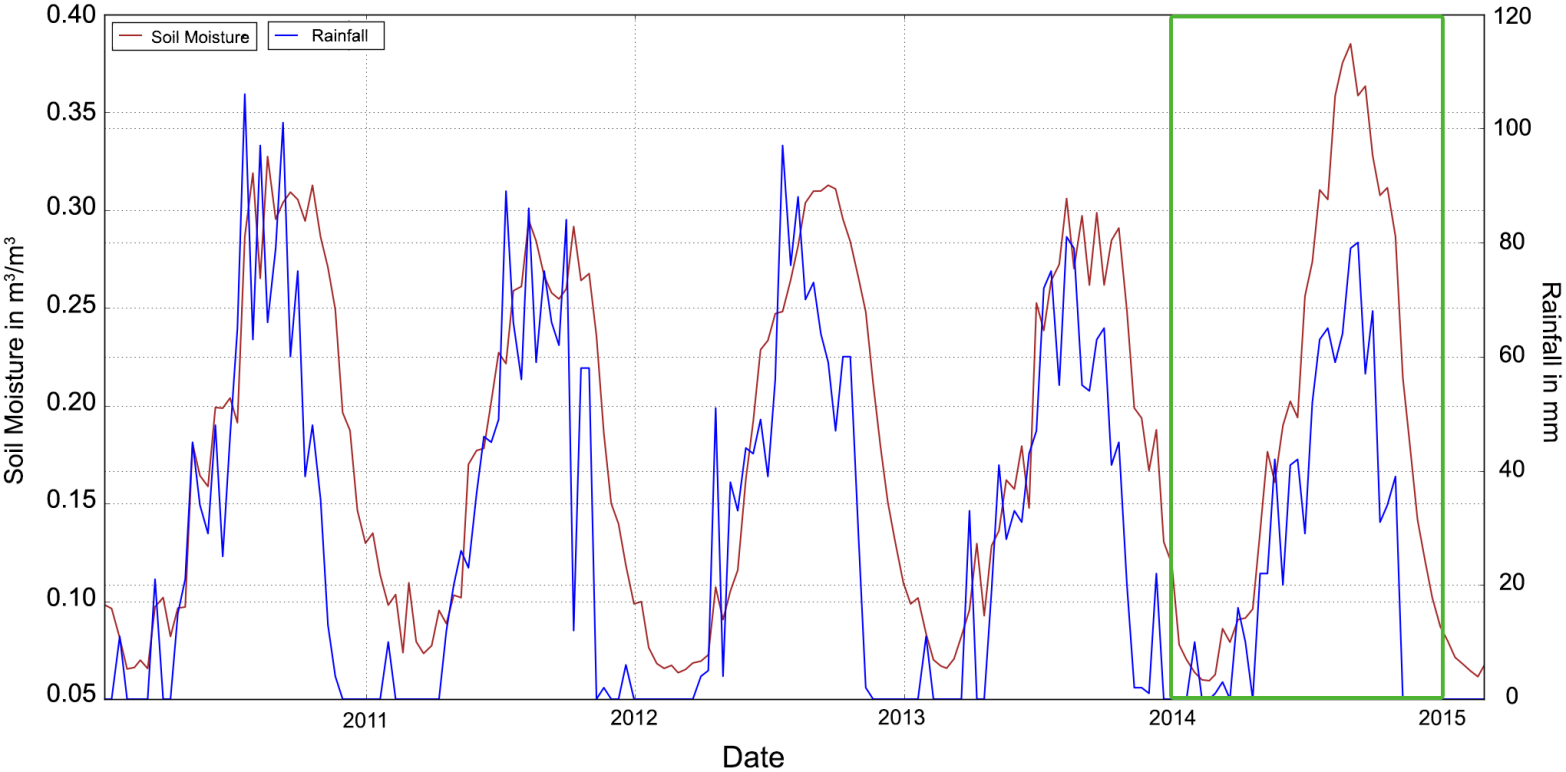
Satellite-derived time series of rainfall (blue) and soil moisture (brown) for the location of Kabo (18.61E/7.69N), covering January 2010 to May 2015 (the green rectangle highlights 2014).

**Fig 5:**
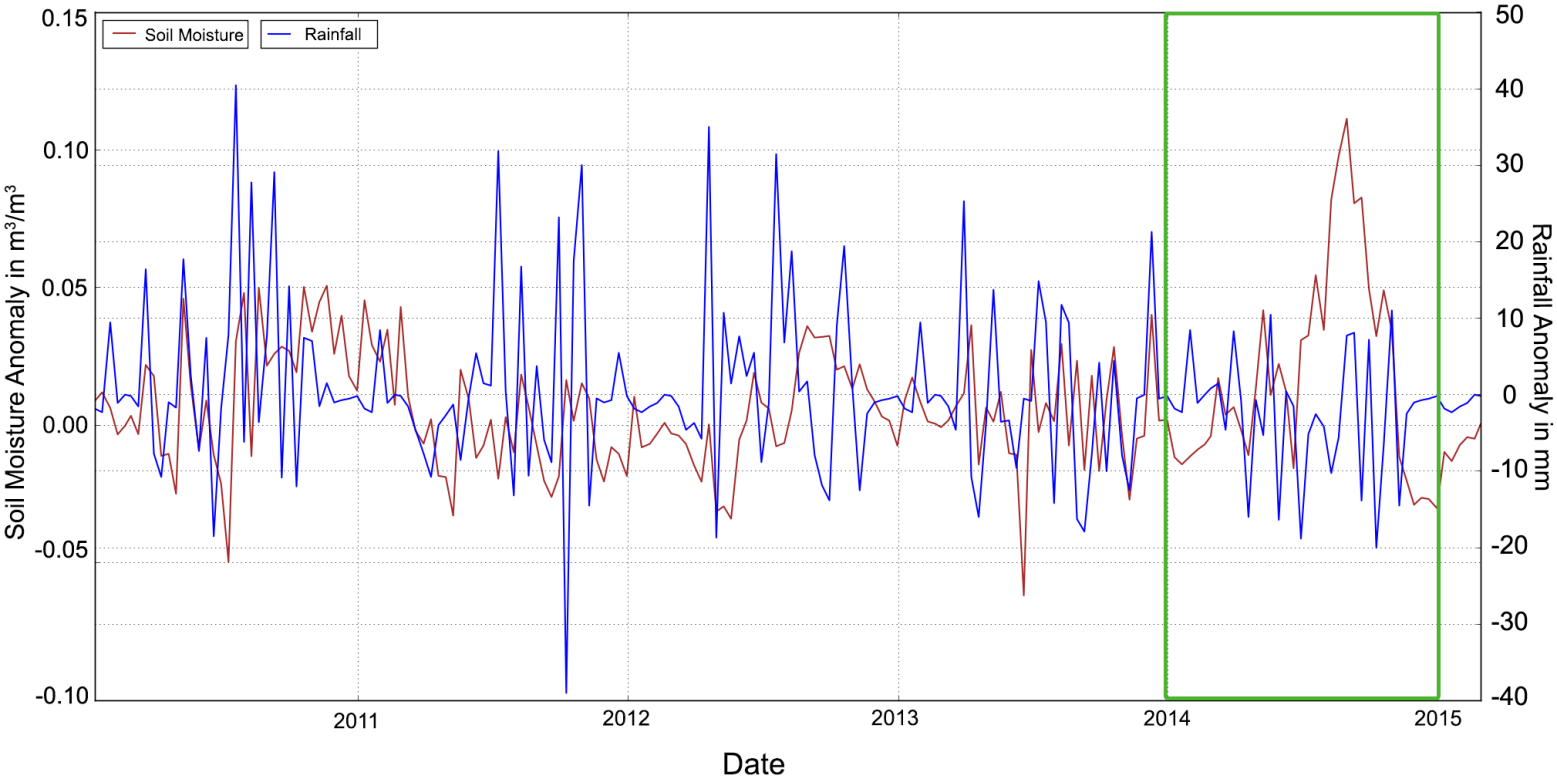
Satellite-derived time series of rainfall (blue) and soil moisture (brown) anomalies for the location of Kabo (18.61E/7.69N), covering January 2010 to May 2015 (the green rectangle highlights 2014).

The satellite-derived time series of rainfall and soil moisture time series (Fig 4) show a good agreement in our region of interest. In combination with the corresponding anomalies for 2010–2015 (Fig 5) they confirm that the food insecurity was rather linked to violent conflicts than to a climatic shock. We only observed a slight soil moisture deficit early in 2014. The positive and negative maxima of the decadal anomalies are +11 mm in November and –20 mm in October for rainfall, respectively, and +0.13 m^3^/m^3^ in September and –0.03 m^3^/m^3^ in December for soil moisture.

**Fig 6:**
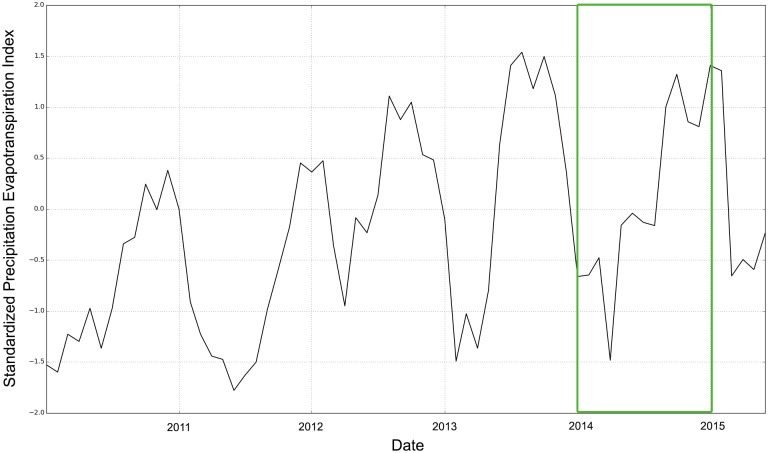
Standardized Precipitation Evapotranspiration Index (SPEI) for the location of Kabo (18.61E/7.69N), covering January 2010 to May 2015 based on a time-scale of 6 months.

Fig 6 shows similar conditions detected by the monthly Standardized Precipitation Evapotranspiration Index (SPEI). Also international and local MSF staff were able to confirm the absence of severe climatic shocks in 2014. However, in the case of a climatic shock, the temporal lag between drought detection and drought impact, or the consequences of a delay in the start of season (SOS) for overall crop production, are vital sources of information for MSF.

## Conclusion

Our study with MSF in the Central African Republic showed that, with appropriate training of local staff, it is possible to substantially increase the efficiency of food security assessments. This was realized through the straightforward adaptation of an Android-based app named SATIDA COLLECT to local conditions (e. g. questions related to people’s diets specific to this region), a quick coverage of large areas, as well as the automated upload of the data collected into a database for advanced processing and data sharing. Despite the lack of a random sampling strategy, which would naturally increase the representativeness of the collected data, our key findings indicate that in May 2015 the interviewed households consumed 0.9 meals per day on average, while the average household size was more than nine people. Despite this fact, children between six and 59 months, the most vulnerable group, were not malnourished. From a total of 923 family members in the assessment 92 had joined the households during the last six months. Again, the movement of IDPs seems to be strongly related to volatile conflict in more Southern regions.

Regarding the process of data collection, in particular, the selection and training of the local staff with respect to the understanding of all questions and logical relationships is crucial. If, for instance, the number of reported births during the last six months is zero while there is one child under the age of six months, this issue must be clarified or corrected (e. g. by repeating the question). In the face of a highly volatile environment due to the ubiquity of armed rebel forces, we strongly recommend to coordinate all activities in the field with the corresponding authorities and heads of districts to minimize any kind of suspicion related to the smart phones.

Based on our experience with the extended questionnaire, one assessment (including the walk from house to house) takes around 20 minutes, which would result in 54 assessments per day, 270 per week (assuming six working hours/day). This way it should easily be possible to cover a representative number of households in Kabo or any other comparable village every week. In any case we suggest to apply a simple, but comprehensible random sampling technique. This strategy requires an early announcement so that people stay at home from agricultural activities and school for as little time as possible.

Analyzing the coupling between socio-economic information, representing inter alia the impact of droughts, and environmental anomalies measured via satellite-derived information, representing drought risk, is important. Also if the current crisis is caused by violent conflict rather than climatic shocks, the impact of droughts on people’s livelihoods can put the already vulnerable system under severe additional pressure, potentially fueling insurgent activities. Remote sensing helps to understand the perennial development of food crises, to relate current to past conditions and to use the potential forecasting capability of environmental information in regions whose food security levels are susceptible to climatic shocks. In the case of Kabo, satellite-derived information about rainfall/soil moisture conditions (Figs 4 and 5) and the Standardized Precipitation Evapotranspiration Index (Fig 6) confirmed that the food insecurity situation 2013/2014 was related to violent conflicts rather than to a climatic shock. We strongly advise to consider the full range of satellite-derived information in the early warning phase of drought-induced food insecurity, ranging from rainfall and soil moisture to evapotranspiration and vegetation-based indicators. In particular with regard to soil moisture we will soon be able to provide observations at a far higher spatial resolution than 0.25 degrees via the European “Sentinel” mission or NASA’s SMAP (Soil Moisture Active/Passive). Creating internal capacities within aid organizations is therefore not enough, because large data volumes and tailored products require dedicated hardware and expertise. Therefore, partnerships between the research community and stakeholders who can apply these solutions in the field are essential. Furthermore, local data collection can also be beneficial for the validation of satellite-derived information, for instance via the use of low-cost soil moisture sensors that transmit the recorded data via a Bluetooth connection.

While the test of SATIDA COLLECT focused on early warning related to food insecurity, discussions with MSF staff lead to additional applications. SATIDA COLLECT can easily be adapted to new applications as outlined in section 2.2. All of them have in common that time and human resources can be saved, as no digitization of paper surveys is needed. In this way, also errors during the process of digitization can be avoided. Possible applications include:

- the evaluation of vaccination efforts (e. g. assessing households that did or did not participate in the vaccination campaign)
- the evaluation of water, sanitation and health (WASH) and food security conditions in refugee camps (ideally one high resolution satellite image is available to number the tents prior to the evaluation)
- replacing the written weekly incidence report (WIR) via standardized forms
- any other application that requires simple, quick and standardized digitization of information

In addition to the basic version of SATIDA COLLECT for assessments on household-level, we developed three other versions within the same application. One version concentrates on village-level assessments and related price developments of staple food. Bumper harvests often lead to a reduction in commodity prices, which in turn results in limited planting that is even more susceptible to climatic shocks [28]. Another version focuses on the admissions to the Inpatient and Ambulatory Therapeutic Feeding Centre (ITFC, ATFC), as well as on widespread diseases (malaria, diarrhea, respiratory infections, measles), comparing current numbers to a reference period in the previous year. All versions will soon be available on the Google Play store free of charge.

In summary, SATIDA COLLECT seems to be the most flexible, efficient and user-friendly data collection tool that MSF have used so far. It is the only tool that facilitates a direct link to satellite-derived information about drought risk. If used for regular assessments, SATIDA COLLECT can help to assess the current levels of food insecurity more realistically and to identify which critical thresholds in satellite-derived datasets and indicators reflect actual drought impacts. Finally, the complementary use of information from satellites and SATIDA COLLECT can support the translation of early warnings into action, reducing the risk of false alarms and strengthening overall disaster preparedness.

## Supporting Information

S1 Table provides a full list of all questions in French (A) that were used for the food security assessment in the Central African Republic and a translation to English (B). (DOCX)
