# Supplementary material for: Food Security Monitoring via Mobile Data Collection and Remote Sensing: Results from the Central African Republic
Source: PLoS One. 2015 Nov 18;10(11):e0142030. doi: 10.1371/journal.pone.0142030 (PMC4651491; doi:10.1371/journal.pone.0142030)
Supplement: S1 Table — (DOCX) [file pone.0142030.s001.docx]

Orange = mandatory questions

Green = optional questions

S1 Table French Questionnaire used for the Assessment (A)

| Données | Réponse | |
| --- | --- | --- |
| S’il vous plaît enregistrez votre position | (Enregistrement du Lieu) | |
| Si l’emplacement ne soi pas enregistré s’il vous plaît indiquez votre position maintenant | Nom de la ville | |
| S’il vous plaît entrez le nom de la maison | Nom du quartier + nom de la famille | |
| Nombre total de personnes au foyer | Entrez un nombre # | |
| Nombre total d’enfants inférieurs à 6 mois au foyer | Entrez un nombre # | |
| Nombre total d’enfants entre 6 mois et 59 mois au foyer | Entrez un nombre # | |
| Nombre total de personnes qui ont on rejoint le foyer au cours des six dernier mois | Entrez un nombre # | |
| Nombre total d’accouchements au cours des six dernier mois | Entrez un nombre # | |
| Nombre total de personnes qui ont quitté le foyer au cours des six dernier mois | Entrez un nombre # | |
| Nombre total d’enfants inférieurs à 5 ans qui ont quitté le foyer au cours des six dernier mois | Entrez un nombre # | |
| Numéro de décès au cours des six dernier mois (total) | Entrez un nombre # | |
| Numéro de décès au cours des six dernier mois (seulement enfants inférieurs à 5 ans) | Entrez un nombre # | |
| Pour CHAQUE ENFANT : œdèmes | - Oui - Non | |
| Si OUI 🡪 NO MUAC (Mid Upper Arm Circumference Measurement Band) |  | |
| Si NO 🡪 MUAC |  | |
| MUAC pour chaque enfant de 6 à 59 mois | - Couleur :   1. Rouge   2. Orange   3. Jaune   4. Vert | |
| Combien de repas le foyer a-t-il consommé dans les dernières 24 heures ? | - 0 par jour - 1 par jour - 2 par jour - 3 par jour (ou plus) | |
| Réserves pour combien de semaines | Entrez un nombre # | |
| Qu’est-ce que le foyer a-t-il consommé dans les dernières 24 heures? | | |
| Céréales | | - Oui - Non |
| Tubercule | | - Oui - Non |
| Légumes secs/lentilles | | - Oui - Non |
| Lait/ produits laitiers | | - Oui - Non |
| Œufs | | - Oui - Non |
| Viande/ fraiche | | - Oui - Non |
| Poisson/ fruits de mer | | - Oui - Non |
| Huile/matière grasse | | - Oui - Non |
| Sucre/Miel | | - Oui - Non |
| Fruits/Légumes | | - Oui - Non |
| Arachides | | - Oui - Non |
| Autres (par ex. épices ou limonade) | | - Oui - Non |
| En cas de distribution générale de rations alimentaire : Quand le foyer a-t-il reçu la dernière distribution ? | | Entrez date (Jour – Mois – Année : XXXXXXXX)  🡪 aussi Mois - Année ou seulement Année possible |
| Mécanismes d’adaptation | | |
| Nourriture moins préférée et moins chère | | - Oui - Non |
| Nourriture et argent emprunté | | - Oui - Non |
| Collecte de nourriture sauvage qu’on ne mangerait pas normalement | | - Oui - Non |
| Récolter des céréales peu mûrs | | - Oui - Non |
| Consommation du stock de graine | | - Oui - Non |
| Nombre réduit de repas par jour | | - Oui - Non |
| Jours sans manger | | - Oui - Non |
| Envoyé les enfants à vivre/manger dans un autre foyer | | - Oui - Non |
| Dépenses réduites pour santé et éducation | | - Oui - Non |
| Vendu des articles du foyer (par ex. ustensiles, couvertures) | | - Oui - Non |
| Vendu des produits agricoles (semences etc. ) | | - Oui - Non |
| Vendu volaille | | - Oui - Non |
| Vendu de petits animaux– chèvres, moutons | | - Oui - Non |
| Vendu grand animaux – bétail | | - Oui - Non |
| D’autres commentaires | | E. g. « Un enfant a décédés pendant l’accouchement » |

S1 Table English Translation of the Questionnaire (B)

| Data to collect | Answer | |
| --- | --- | --- |
| Please track your location | GPS tracking | |
| If you cannot track your location please enter your current position | Name of the town | |
| Please enter the name of the household | Name of the district + name of the family | |
| Number of people in the household | Insert number # | |
| Number of children under 6 months in the household | Insert number # | |
| Number of children between 6 and 59 months in the household | Insert number # | |
| Number of people that joined the household during the last six months | Insert number # | |
| Number of births during the last six months | Insert number # | |
| Number of people that left the household during the last six months | Insert number # | |
| Number of children under the age of 5 that left the household during the last six months | Insert number # | |
| Number of deaths during the last six months (total) | Insert number # | |
| Number of deaths (only children under the age of 5) during the last six months | Insert number # | |
| For EACH CHILD (6-59 months) check for oedema | - Yes - No | |
| If YES 🡪 Don’t check MUAC |  | |
| If NO 🡪 Check MUAC |  | |
| MUAC of children 6-59 months (one measurement per child) | - Colour:   1. Red   2. Orange   3. Yellow   4. Green | |
| How many meals did the household consume during the last 24 hours | - 0 per day - 1 per day - 2 per day - 3 per day (or more) | |
| Number of weeks that the food reserves will last | Insert number # | |
| What did the household consume during the last 24 hours? | | |
| Cereals | | - Yes - No |
| Roots/ tubers | | - Yes - No |
| Pulses/ lentils | | - Yes - No |
| Milk/ milk products | | - Yes - No |
| Eggs | | - Yes - No |
| Meat/ offal/ bowels | | - Yes - No |
| Fish/ seafood | | - Yes - No |
| Oil/ fat | | - Yes - No |
| Sugar/ honey | | - Yes - No |
| Fruits/ vegetables | | - Yes - No |
| Peanuts | | - Yes - No |
| Other (e.g. spices, soda) | | - Yes - No |
| In case of a GFD (General Food Distribution) in the area: When did the household receive the last GFD? | | Insert Date (Day – Month- Year: XXXXXXXX)  🡪 also Month – Year or only Year possible |
| Coping mechanisms | | |
| Rely on less preferred and less expensive food | | - Yes - No |
| Borrowed food, money by relatives/friend | | - Yes - No |
| Gathered wild food that you would normally not eat | | - Yes - No |
| Harvesting immature crops | | - Yes - No |
| Consumed seed stock held for next season | | - Yes - No |
| Reduced meal portions | | - Yes - No |
| Reduced the number of meals per day | | - Yes - No |
| Skipped days without eating | | - Yes - No |
| Sent children to live or eat in another household | | - Yes - No |
| Reduced expenditures on health and education | | - Yes - No |
| Sold household articles (e.g. utensils, blankets) | | - Yes - No |
| Sold household agricultural tools, seeds, etc | | - Yes - No |
| Sold household poultry | | - Yes - No |
| Sold small animals – goats, sheep | | - Yes - No |
| Sold large animals – cattle (cows) | | - Yes - No |
| Other comments | | For instance: “One child died during birth”. |
